# Whole Transcriptome Analysis of Notochord-Derived Cells during Embryonic Formation of the Nucleus Pulposus

**DOI:** 10.1038/s41598-017-10692-5

**Published:** 2017-09-05

**Authors:** Sun H. Peck, Kendra K. McKee, John W. Tobias, Neil R. Malhotra, Brian D. Harfe, Lachlan J. Smith

**Affiliations:** 1 Department of Neurosurgery, Perelman School of Medicine, University of Pennsylvania, Philadelphia, Pennsylvania USA; 2 Department of Orthopaedic Surgery, Perelman School of Medicine, University of Pennsylvania, Philadelphia, Pennsylvania USA; 3 Department of Molecular Genetics and Microbiology, The Genetics Institute, College of Medicine, University of Florida, Gainesville, Florida USA; 4 Penn Genomics Analysis Core, University of Pennsylvania, Philadelphia, Pennsylvania USA

## Abstract

Recapitulation of developmental signals represents a promising strategy for treating intervertebral disc degeneration. During development, embryonic notochord-derived cells (NDCs) are the direct progenitors of cells that populate the adult nucleus pulposus (NP) and are an important source of secreted signaling molecules. The objective of this study was to define global gene expression profiles of NDCs at key stages of embryonic disc formation. NDCs were isolated from *Shh-cre*;ROSA:YFP mice at embryonic day 12.5 and postnatal day 0, representing opposite ends of the notochord to NP transformation. Differences in global mRNA abundance across this developmental window were established using RNA-Seq. Protein expression of selected molecules was confirmed using immunohistochemistry. Principal component analysis revealed clustering of gene expression at each developmental stage with more than 5000 genes significantly differentially expressed between E12.5 and P0. There was significantly lower mRNA abundance of sonic hedgehog pathway elements at P0 vs E12.5, while abundance of elements of the transforming growth factor-beta and insulin-like growth factors pathways, and extracellular matrix components including collagen 6 and aggrecan, were significantly higher at P0. This study represents the first transcriptome-wide analysis of embryonic NDCs. Results suggest signaling and biosynthesis of NDCs change dramatically as a function of developmental stage.

## Introduction

The intervertebral discs are the partially movable joints that consecutively connect the vertebrae of the spine. Each intervertebral disc is comprised of three substructures: centrally, the proteoglycan-rich nucleus pulposus (NP); peripherally, the fibrocartilaginous annulus fibrosus; and superiorly and inferiorly, two endplates of hyaline cartilage^1–4^. These three substructures act synergistically to absorb and transfer compressive forces and facilitate complex motion of each intervertebral joint. Embryonic formation of the intervertebral discs centers on the notochord, a highly specialized, mesoderm-derived, transient, midline structure that is present in all chordates during early development^5^. The notochord initially serves as a primitive structural axial skeleton in the developing embryo and as the main signaling center for providing patterning information for surrounding tissues during early development through the regulation of secreted molecular factors^6–11^. In higher vertebrates, this singular structure eventually transforms into distinct NPs within the intervertebral discs during late stages of embryonic development^12–14^. In mice, this transformation occurs across an 8 day window commencing at embryonic day 12.5^15^. The molecular mechanisms that regulate transformation of the notochord into the NPs remain poorly understood.

Fate mapping studies in mice have demonstrated that all cells in the adult NP are derived from the notochord^16, 17^. In humans, the phenotype of NP cells changes markedly during growth and aging^18–20^ and by skeletal maturity, these cells have lost the majority of their notochordal characteristics and assume physical and molecular characteristics that more closely resemble those of articular cartilage chondrocytes^21^. However, molecular profiling studies have demonstrated that these adult NP cells do continue to maintain high expression of some notochordal markers^22^.

Degeneration of the intervertebral discs, which is ubiquitous amongst the aging adult population, is strongly implicated as a cause of low back pain, a condition that will affect around 85% of all people at some point in their lifetime and costs over $100 billion annually in the United States^23–26^. Disc degeneration is a cascade of cellular, structural, and biomechanical changes that is closely linked with aging^23, 27^. The earliest manifestations of disc degeneration typically occur in the NP, where reduced proteoglycan content compromises mechanical function leading to progressive structural deterioration of the entire intervertebral joint. Current treatments for low back pain include physical therapy, steroid injections, or where surgery is warranted, spinal fusion or artificial total disc replacement^28^. These treatments, however, seek to manage symptoms without maintaining or restoring native disc structure or biomechanical function. As such, a key focus of current research efforts is to develop new biological treatment strategies that can both address symptoms and regenerate native disc tissue.

An attractive strategy with high promise for long term reconstitution of healthy disc tissue is cell-based regeneration using therapeutic cell types such as mesenchymal stem cells (MSCs) or induced pluripotent stem cells (IPSCs)^29, 30^. One of the major impediments to successful stem cell-driven disc regeneration is the requirement for delivered cells to regenerate multiple tissues, each with distinct architecture and composition, and each comprised of cells with disparate developmental lineages.

Given the established function of the notochord as a source of secreted signaling molecules that regulate embryonic disc formation, there is intense interest in identifying notochordal cell-secreted factors and applying them to develop improved therapeutic strategies for disc regeneration^31^. Previous studies suggest that such factors may potentiate anabolic disease-modifying effects^32–36^. As the notochord is a transient structure and disappears in early development, harvesting notochordal cells for direct therapeutic use for disc regeneration is not feasible; however, improved understanding of embryonic NP formation may enable recapitulation of important developmental signals that are necessary for the formation of the NP and its proteoglycan-rich extracellular matrix (ECM).

The overall objective of this study was to undertake a global analysis of the molecular regulation of embryonic disc formation by mapping the transcriptome of notochord-derived cells (NDCs) at developmental stages representing opposite ends of the notochord to NP transformation. In our analysis, we focused on signaling pathways with established roles regulating skeletal patterning and growth, cell differentiation, and extracellular matrix deposition. Additionally, we examined changing expression levels of key ECM molecules and putative NP cell-specific markers as previously defined in the literature^37^.

## Materials and Methods

### Animals and Tissue Collection

**Figure 1:**
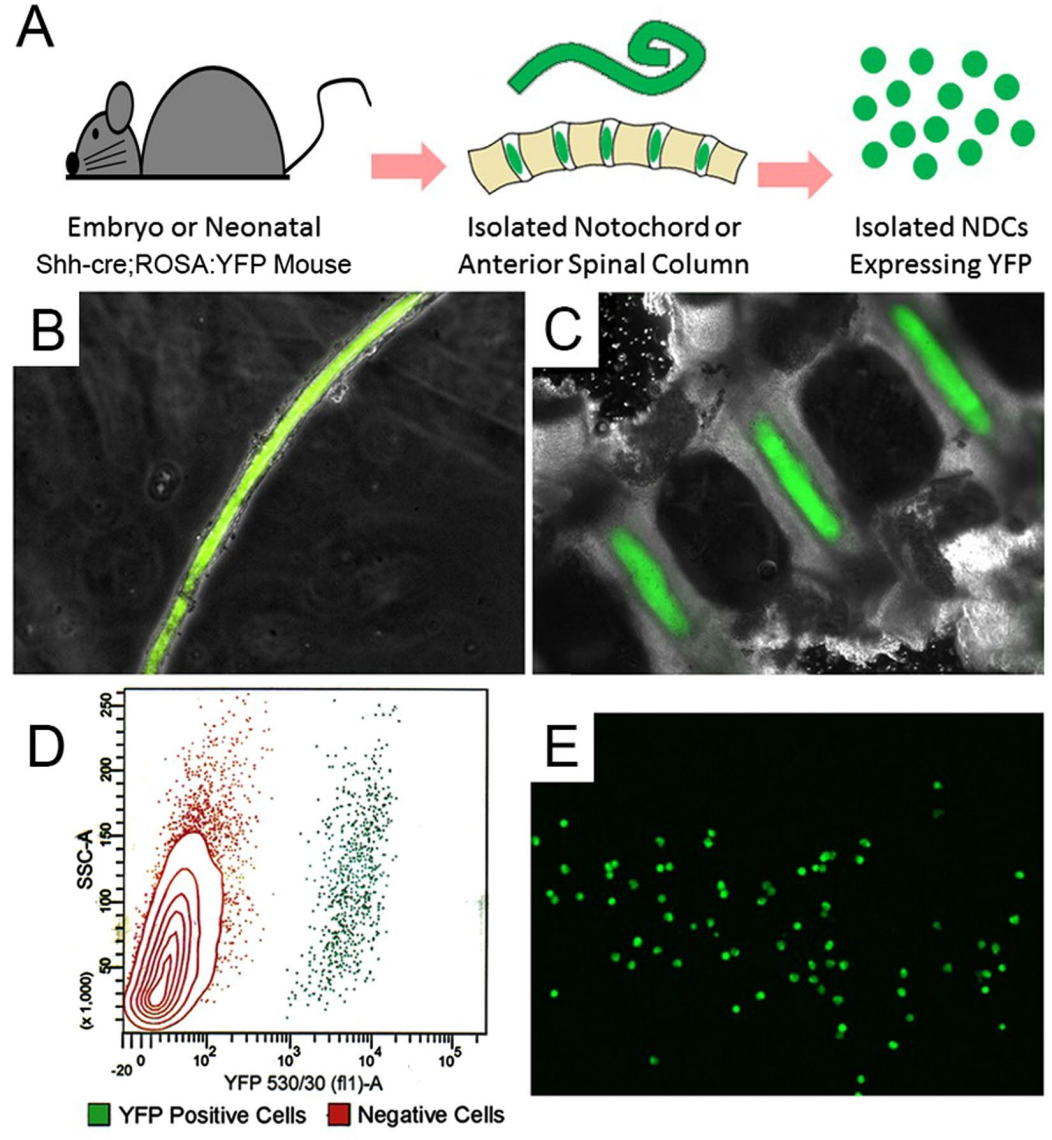
Isolation of YFP-positive NDCs from *Shh-cre*;ROSA:YFP mice. (**A**) Cell isolation schematic. (**B**) YFP-positive E12.5 notochord. (**C**) YFP-positive nucleus pulposi in a P0 spine. (**D**) FACS plot for P0 NDC sort. (**E**) Isolated YFP-positive NDCs.

For these studies, we used the *Shh-cre*;ROSA:YFP mouse model, previously established and shown to express YFP in SHH-expressing notochord cells and their progeny at any developmental stage^16^ (Fig. 1). Animals were raised under NIH guidelines for the care and use of animals in research, and all experimental studies were carried out with approval from the Institutional Animal Care and Use Committee of the University of Florida. Animals were euthanized at two developmental stages, embryonic day 12.5 (E12.5) and postnatal day 0 (P0), representing opposite ends of the notochord to NP transformation^15^. Euthanasia of pregnant mothers was achieved via cervical dislocation followed by tracheotomy, after which the E12.5 embryos were collected and placed into nuclease-free PBS. P0 pups were euthanized by decapitation and placed into nuclease-free PBS. Biological replicates (n = 4 at both time points) each consisted of pooled embryos or pups (~6) from a single litter.

Following euthanasia, for E12.5 embryos, whole, intact notochords (Fig. 1B) were harvested under a dissecting microscope, any extraneous adjoining tissue carefully removed, immediately placed into TRIzol (Ambion; Austin, TX) and frozen. For P0 pups, anterior vertebral columns (Fig. 1C) were harvested, and each disc space in the lumbar and thoracic regions was carefully bisected axially under a dissecting microscope on ice to expose the NPs (Schematic, Fig. 1A). Gentle mechanical agitation in PBS buffer with 25 mM HEPES and 0.1% BSA to minimize cell stickiness was then used to dissociate NP cells from the surrounding tissue, and the resulting cell mixture was strained through a 50 µm mesh filter. Enzymatic cell isolation was specifically avoided as it has been shown that exposure to enzymes such as collagenase can confound global mRNA expression patterns^38^. The collected cells underwent fluorescence assisted cell sorting (FACS) in the PBS buffer described above and YFP-positive NDCs (~10,000–30,000 cells per litter) were collected directly into TRIzol LS (Ambion; Austin, TX) and frozen (Fig. 1D,E).

### RNA Extraction, Library Preparation, and Whole-transcriptome Sequencing

Whole-transcriptome sequencing (RNA-Seq) is a powerful tool that can accurately quantify global mRNA expression in many samples in parallel, which allows for direct comparisons of expression levels of many genes-of-interest at once. Another advantage of RNA-Seq is the ability to measure transcript levels over a large range of expression with limited background noise and signal saturation, which allows for detection of rare and lowly expressed transcripts.

RNA was extracted using serial TRIzol-chloroform extractions, then in-column treated with RNase-free DNase (Qiagen; Valencia, CA) on Direct-zol RNA MiniPrep columns (Zymo Research; Irvine, CA) and eluted following the manufacturer’s protocols. Separate aliquots of extracted RNA samples were stored at −80 °C for either RNA quality analysis or RNA-Seq library preparation in order to reduce the number of freeze-thaw cycles.

The quality of each RNA sample was verified using an Agilent BioAnalyzer and RNA 6000 Pico Kit (Santa Clara, CA). Only high quality total RNA (RNA integrity number >7) was used to prepare RNA-Seq libraries. Libraries were prepared using the TotalScript Kit (Illumina; San Diego, CA), which has been optimized for use in synthesizing RNA-Seq libraries from low amounts of total starting RNA (<5 ng). Single-end, 100-base pair sequencing was performed using the Illumina HiSeq 2500 platform at the Next Generation Sequencing Core of the University of Pennsylvania. Results were aligned to the mouse genome build GRCm38.p3 (NCBI) and genomic features annotated from the M4 build from Gencode (www.gencodegenes.org).

### RNA-Seq Analysis

Fastq files containing raw sequence and quality scores were mapped to the mouse genome (GRCm38) using the STAR aligner (https://www.ncbi.nlm.nih.gov/pubmed/23104886). Genomically mapped reads were counted against reference genes as annotated in version M4 from Gencode (version M4, https://www.ncbi.nlm.nih.gov/pubmed/26187010) using htseq-count (https://academic.oup.com/bioinformatics/article-lookup/doi/10.1093/bioinformatics/btu638). DESeq2^39^ was used to generate regularized log2-transformed expression values for all genes in all samples, which were visualized to assess inter-sample variability using Principal Components Analysis (PCA) as implemented in Partek Genomics Suite (v6.6, Partek, Inc., St. Louis, MO). DESeq2 was used to calculate statistics for differential expression between the E12.5 and P0 groups. Genes were considered significant if they showed a false-discovery-rate adjusted p-value of <0.05. We carried out pathway analysis (Ingenuity Pathway Analysis; Qiagen, Valencia, CA), through which we applied a structured approach to our analysis of specific pathways and molecules, focusing on signaling pathways with well-defined roles in tissue patterning and growth including cell migration, proliferation and differentiation, families of extracellular matrix molecules that are important for tissue assembly, structure and mechanical function, and previously identified markers of the NP cell phenotype.

### Histology and Immunohistochemistry

Immunohistochemical detection of protein expression was undertaken for selected molecules identified through RNA-Seq. Representative samples of whole embryos (E12.5) or anterior spinal columns (P0) from five litters were placed into 4% paraformaldehyde overnight. Following fixation, P0 spines were gently decalcified in 0.5 M EDTA at pH 8.0 for 2 days. E12.5 whole embryos and P0 spines were then processed into paraffin and sectioned at 8 µm. For each antibody, E12.5 and P0 samples were stained in parallel to standardize the timing of each step throughout the protocol. Antigen retrieval was carried out on rehydrated sections using a heat-mediated technique in a 95 °C buffer bath of 10 mM sodium citrate with 0.05% Tween 20, pH 6.0 for 20 minutes. Sections were permeabilized in TBS +0.025% Triton X-100 (TBS-T) and blocked using Background Buster (Accurate Chemical & Scientific Corporation; Westbury, NY) for 30 minutes at room temperature. Sections were then incubated with primary antibodies against sonic hedgehog (SHH), transforming growth factor beta 1 (TGF-β1), insulin-like growth factor-1 (IGF-1), aggrecan (ACAN), and collagens 1, 2, and 6 (COL1, COL2, COL6) (primary antibody information is provided in Supplemental Table S1) diluted in Background Buster overnight at 4 °C. Slides were rinsed twice, 5 minutes each in TBS-T, then incubated in 0.3% H_2_O_2_ in TBS for 15 minutes to suppress endogenous peroxidase activity. Slides were rinsed three times, 2 minutes each with TBS. Antibody staining was visualized using the SuperPicture Polymer Detection kit, DAB, rabbit (ThermoFisher Scientific; Waltham, MA) using the manufacturer’s protocol. Slides were rinsed well in diH_2_O, and then hematoxylin QS (Vector Laboratories; Burlingame, CA) was applied for 30 seconds. Slides were then rinsed with tap H_2_O, dehydrated and cleared, and coverslipped with Permount Mounting Medium (ThermoFisher Scientific; Waltham, MA). Slides were imaged under bright field light microscopy (Eclipse 90i; Nikon; Tokyo, Japan). As negative controls, sections without primary antibodies were treated in parallel with the same protocol, except with only Background Buster during the overnight primary antibody incubation (representative images, Supplemental Fig. S1). Additionally, to assess overall tissue composition and morphology, sections were double-stained with either Alcian blue and picrosirius red (ABPR) for glycosaminoglycans (GAG) and collagen, respectively, or hematoxylin and eosin (H&E) to demonstrate cellularity and imaged under bright field light microscopy.

### Data Availability Statement

The datasets generated and analyzed during this study are available in the NCBI Gene Expression Omnibus (GEO) repository, accession number: GSE100934.

## Results

### Morphological Appearance of the Spinal Column at E12.5 and P0

**Figure 2:**
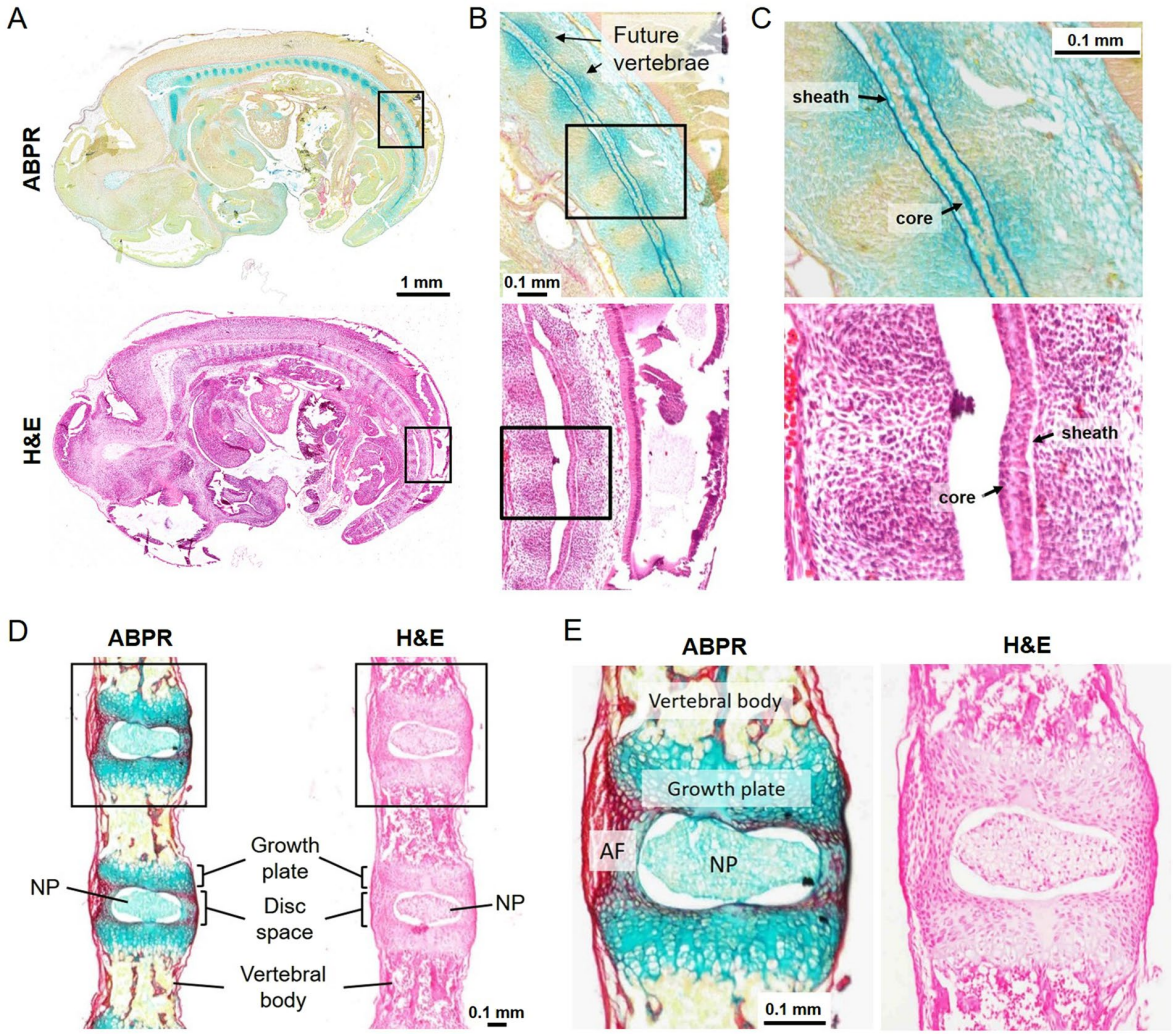
Overall morphological characteristics of E12.5 embryos and P0 spines. (**A**) ABPR and H&E stained E12.5 embryo sections. (**B**) Black box insets of Fig. 2A. Mesenchymal condensations indicating locations of future vertebrae are labeled. (**C**) Black box insets of Fig. 2B. GAG-rich, relatively acellular outer sheath and inner core of notochord are labeled. (**D**) ABPR and H&E stained P0 spine sections showing key anatomical features. (**E**) Black box insets of Fig. 2D.

Histological examination confirmed the expected morphological characteristics of the vertebral column at each developmental stage (Fig. 2). At E12.5, the notochord was observed as a single discrete structure spanning the length of the axial skeleton and was encased in a GAG-rich sheath (Fig. 2A–C). Mesenchymal condensations, representing future vertebral bodies, stained positively for GAG (Fig. 2B,C). The central region of the notochord contained a core that stained intensely for GAGs and appeared relatively acellular (Fig. 2C). At P0, NPs were fully formed within the intervertebral discs (Fig. 2D). The population of cells within the NPs at P0 appeared morphologically homogeneous (Fig. 2E, H&E staining). The lamellar architecture of the annulus fibrosus of the discs was established, and in the vertebrae, primary ossification was complete with secondary ossification yet to commence.

### RNA-Seq and Pathway Analysis

**Figure 3:**
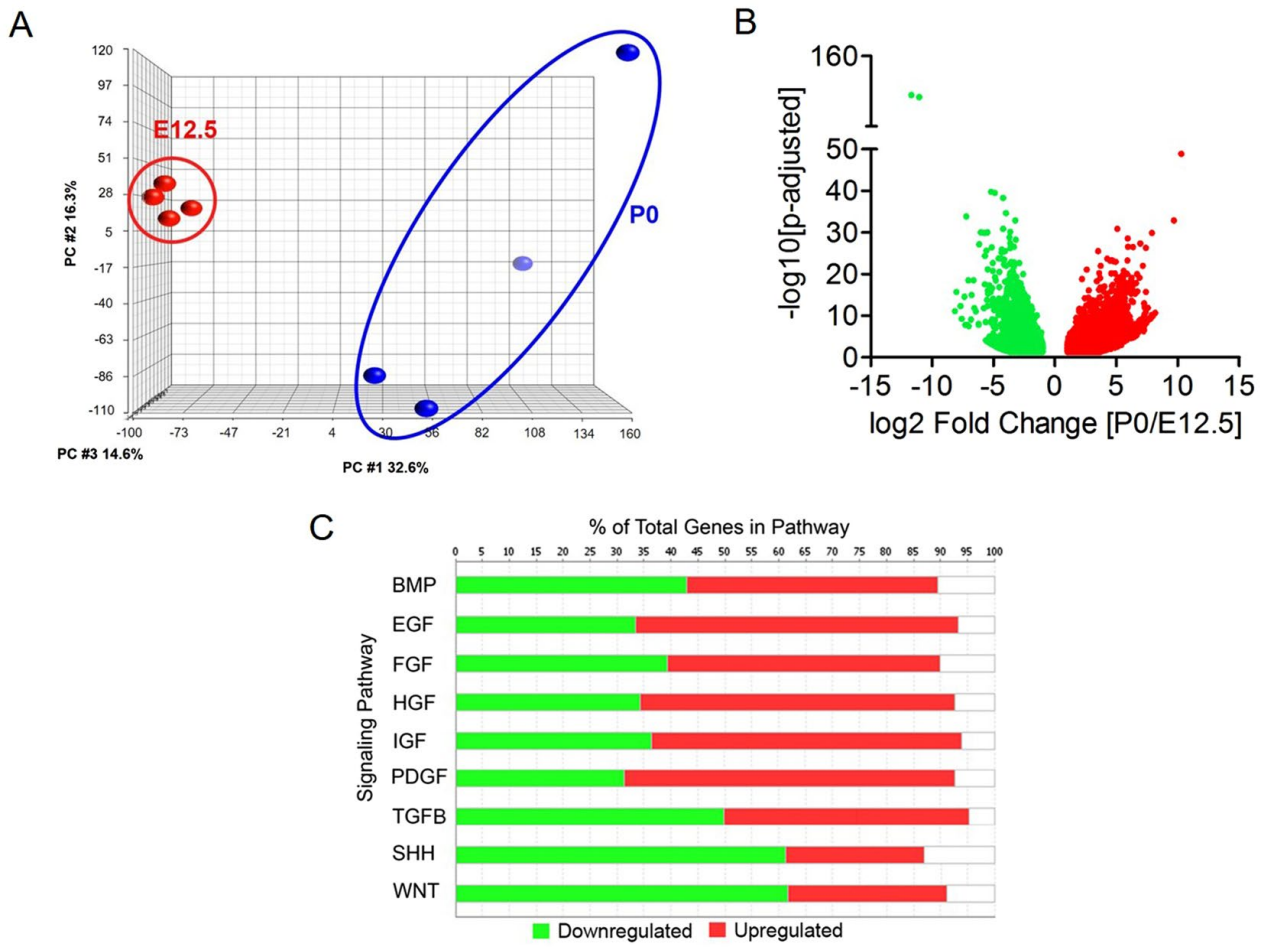
Global RNA-Seq analysis. (**A**) Principle component analysis of RNA-Seq data. (**B**) Volcano plot showing log2-fold change of mRNA abundance as a ratio of P0 expression to E12.5 expression. (**C**) Pathway analysis of RNA expression data showing percent of total genes significantly differentially expressed at P0 compared to E12.5 for key developmental pathways. N = 4.

Principal component analysis (PCA) revealed clustering of samples at E12.5 and P0 indicating clear effects of developmental stage on global mRNA abundance (Fig. 3A). There were 5015 genes significantly differentially expressed with fold-changes greater than 2 between the two developmental stages: 2022 genes were upregulated and 2993 genes were downregulated at P0 compared to E12.5 (Fig. 3B). Pathway analysis revealed signaling pathways previously established as important regulators of embryonic tissue morphogenesis as exhibiting significant differential expression at P0 compared to E12.5. These included the sonic hedgehog (Shh), transforming growth factor-beta (TGF-β), and insulin-binding growth factor (IGF) signaling pathways, amongst others (Fig. 3C).

### Validation of Secreted Signaling Molecules, Pathway Elements, and Targets

**Figure 4:**
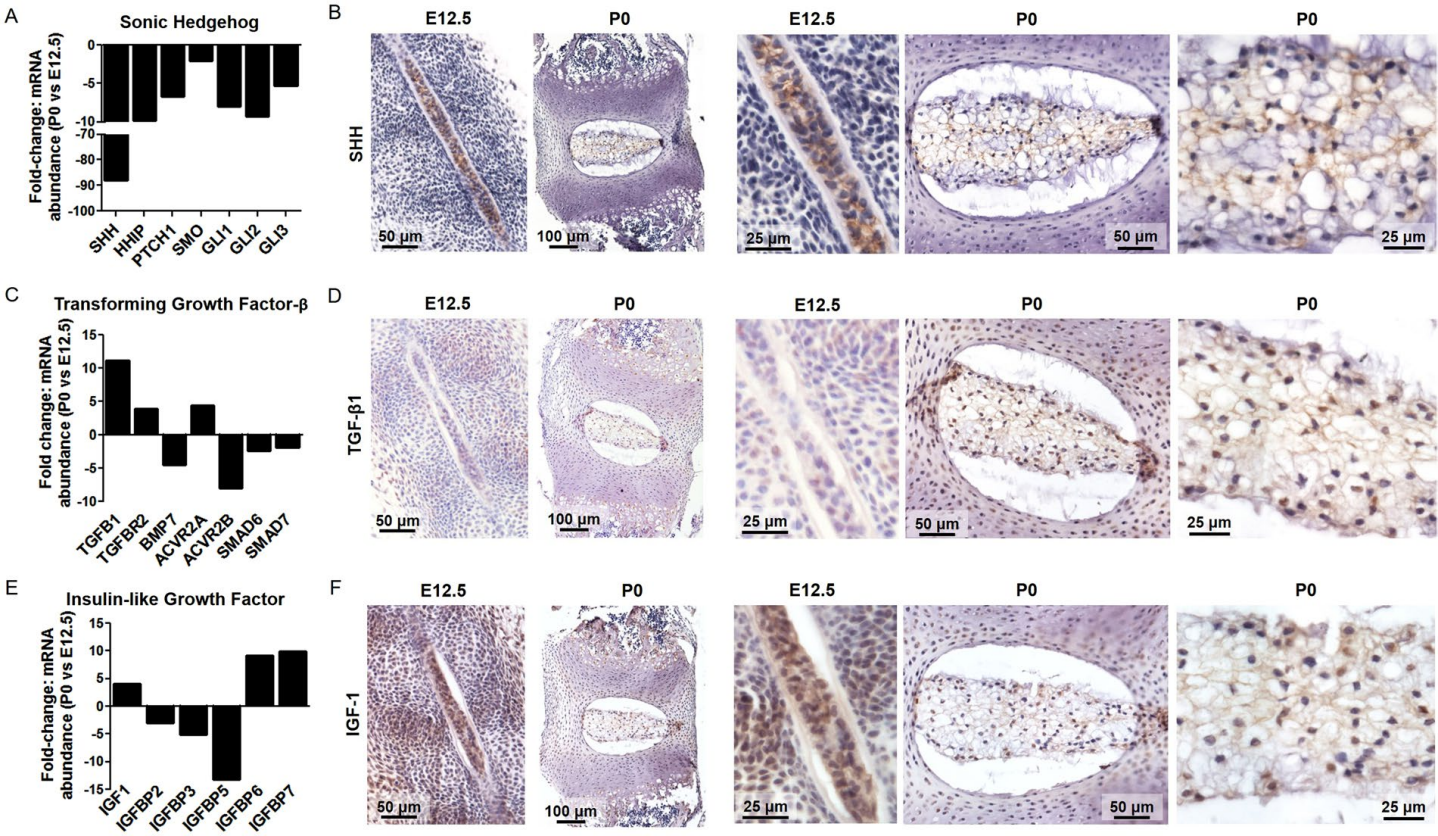
Fold-change in mRNA expression of P0 vs E12.5 NDCs for selected developmental pathways and immunohistochemical analysis of component growth factors at E12.5 and P0. (**A**) Fold-change in mRNA expression of Shh signaling pathway elements showing significant downregulation at P0. (**B**) SHH protein staining showing intense positive staining of the notochord at E12.5 and diffuse staining of the NP at P0. (**C**) Fold-change in mRNA expression of TGF-β pathway elements showing significant upregulation of both *Tgfb1* and *Tgfbr2* at P0. (**D**) TGF-β1 protein staining showing few positively stained notochord cells at E12.5, and widespread positive staining of NP cells and diffuse NP ECM staining at P0. (**E**) Fold-change in mRNA expression of IGF pathway elements showing significant upregulation of *Igf1* at P0. (**F**) IGF-1 protein staining showing positive staining of the notochord at E12.5, and widespread positive staining of NP cells and diffuse NP ECM staining at P0. N = 4; all fold changes p < 0.05, P0 compared to E12.5, analyzed by DESeq2 and adjusted for false discovery rate. Refer to Fig. 2 for annotation of structural regions.

There was significantly lower mRNA abundance of Shh pathway activators including ligand (*Shh*), receptors (Patched1 (*Ptch1*); Smoothened (*Smo*)), and transcription factors (*Gli1*, *2*, *3*) (Table 1, Fig. 4A) at P0 when compared to E12.5. *Shh* mRNA showed the largest overall change with 87.8-fold lower abundance at P0 compared to E12.5. Immunohistochemistry was performed to confirm the downregulation of SHH protein expression at P0 compared to E12.5. In line with RNA-Seq data, there was intense SHH-positive staining in the notochord at E12.5 and light diffuse SHH staining in the NP at P0 (Fig. 4B).

**Table 1:** Fold-change in mRNA expression of Shh pathway genes at P0 compared to E12.5.

Gene	Name	Fold Change in Expression P0 vs E12.5	p value
*Gli1*	GLI family zinc finger 1	−7.99	1.05E-18
*Gli2*	GLI family zinc finger 2	−9.23	1.00E-12
*Gli3*	GLI family zinc finger 3	−5.27	5.35E-07
*GliS1*	GLIS family zinc finger 1	−7.43	1.09E-07
*GliS2*	GLIS family zinc finger 2	−3.13	2.31E-04
*Hhip*	Hedgehog interacting protein	−9.86	6.54E-15
*Ptch1*	Patched 1	−6.72	3.34E-17
*Ptch2*	Patched 2	−5.05	1.36E-04
*Shh*	Sonic Hedgehog	−87.82	8.84E-12
*Smo*	Smoothened	−2.03	5.07E-03
*Sufu*	SUFU negative regulator of hedgehog signaling	−2.05	1.89E-03

N = 4, Differential expression analyzed by DESeq2 and p values adjusted for false discovery rate.

Around 95% of genes associated with the TGF-β pathway were differentially expressed at P0 compared to E12.5 (Fig. 3C), including 11.2 and 3.9-fold higher mRNA abundance of the secreted ligand *Tgfb* *1* and its receptor *Tgfbr2*, respectively (Table 2, Fig. 4C). Immunohistochemistry revealed widespread TGF-β1 positive cells in the mesenchymal progenitor population surrounding the notochord at E12.5 and TGF-β1 staining of relatively few cells within the notochord itself (Fig. 4D). At P0, there was widespread staining of cells within the NP and diffuse staining within the ECM (Fig. 4D). Around 93% of IGF pathway elements exhibited differential expression at P0 compared to E12.5 (Fig. 3C), including a 4.12-fold mRNA increase in the secreted ligand *Igf1* as well as 9.11-fold and 9.92-fold increases in IGF binding proteins 6 and 7, respectively (Table 3, Fig. 4E). Immunohistochemistry for IGF-1 protein expression revealed positive staining at E12.5 within the notochord as well as in the surrounding mesenchymal progenitor population (Fig. 4F). Similarly to TGF-β1, there was widespread IGF-1 staining of cells in the NP and diffuse IGF-1 staining of the ECM at P0 (Fig. 4F).

**Table 2:** Fold-change in mRNA expression of TGF-β pathway genes at P0 compared to E12.5.

Gene	Name	Fold Change in Expression	p value
		P0 vs E12.5	
*Acvr2a*	activin A receptor type IIA	4.39	1.71E-04
*Acvr2b*	activin A receptor type IIB	−7.92	3.35E-16
*Bmp1*	Bone morphogenetic protein 1	−2.04	1.28E-02
*Bmp2k*	BMP2-inducible protein kinase	2.66	7.52E-03
*Bmp3*	Bone morphogenetic protein 3	−5.93	2.35E-08
*Bmp4*	Bone morphogenetic protein 4	−3.13	1.98E-05
*Bmp7*	Bone morphogenetic protein 7	−4.45	3.10E-08
*Bmpr1b*	Bone morphogenetic protein receptor type-1B	−4.05	9.93E-05
*Fst*	Follistatin	−3.79	9.09E-07
*Grb2*	growth factor receptor bound protein 2	2.64	1.32E-04
*Grem1*	Gremlin 1	3.77	1.11E-03
*Nfkb1*	nuclear factor of kappa light polypeptide gene enhancer in B-cells 1	3.49	2.63E-07
*Nfkb2*	nuclear factor of kappa light polypeptide gene enhancer in B-cells 2 (p49/p100)	3.28	8.34E-04
*Nog*	noggin	−28.36	3.52E-19
*Smad5*	SMAD family member 5	−2.24	4.29E-06
*Smad6*	SMAD family member 6	−2.39	7.15E-03
*Smad7*	SMAD family member 7	−1.89	3.37E-02
*Sostdc1*	sclerostin domain containing 1	9.09	1.83E-10
*Tfe3*	transcription factor binding to IGHM enhancer 3	2.04	5.43E-03
*Tgfb1*	Transforming growth factor beta 1	11.18	2.38E-11
*Tgfbr2*	Transforming growth factor beta receptor 2	3.92	2.86E-08
*Znf423*	zinc finger protein 423	−9.70	3.89E-23

N = 4, Differential expression analyzed by DESeq2 and p values adjusted for false discovery rate.

**Table 3:** Fold-change in mRNA expression of IGF pathway genes at P0 compared to E12.5.

Gene	Name	Fold Change in Expression P0 vs E12.5	p value
*Elk1*	ELK1, member of ETS oncogene family	−1.86	1.61E-02
*Fos*	FBJ murine osteosarcoma viral oncogene homolog	23.33	4.50E-12
*Foxo1*	forkhead box O1	1.82	3.55E-02
*Grb10*	growth factor receptor bound protein 10	−3.99	6.37E-07
*Grb2*	growth factor receptor bound protein 2	2.64	1.32E-04
*Igf1*	insulin-like growth factor 1	4.12	2.64E-03
*Igf2*	insulin-like growth factor 2	−10.88	1.45E-15
*Igfbp2*	insulin-like growth factor binding protein 2	−3.04	1.09E-02
*Igfbp3*	insulin-like growth factor binding protein 3	−5.06	7.78E-06
*Igfbp5*	insulin-like growth factor binding protein 5	−13.16	3.92E-23
*Igfbp6*	insulin-like growth factor binding protein 6	9.11	2.15E-04
*Igfbp7*	insulin-like growth factor binding protein 7	9.92	1.05E-09
*Igf2bp1*	insulin-like growth factor 2 mRNA binding protein 1	−9.22	5.53E-29
*Igf2bp2*	insulin-like growth factor 2 mRNA binding protein 2	−12.52	7.07E-31
*Igf2bp3*	insulin-like growth factor 2 mRNA binding protein 3	−1.87	2.46E-03
*Igf2os*	insulin-like growth factor 2, opposite strand	−10.24	2.11E-09
*Igf2r*	insulin-like growth factor 2 receptor	−3.18	3.43E-04
*Irs1*	insulin receptor substrate 1	−3.22	1.04E-03
*Jak1*	Janus kinase 1	2.77	3.88E-05
*Jak2*	Janus kinase 2	1.98	3.85E-03
*Stat3*	signal transducer and activator of transcription 3 (acute-phase response factor)	2.89	6.16E-06

N = 4, Differential expression analyzed by DESeq2 and p values adjusted for false discovery rate.

Over 90% of Wnt signaling pathway elements exhibited significant differential mRNA expression at P0 compared to E12.5 (Fig. 3C), including pathway ligands *Wnt 1*, *3*, *6*, *11*, *5a*, *7a*, *7b* and *9b* (all lower abundance at P0) and *Wnt16* (higher abundance at P0), modulators such as *Sfrps* (secreted frizzled-related proteins), and downstream target genes, *Axin2*, *Cd44*, and *Myc* (Supplemental Table S2). Additional differential gene expression of molecules in other key signaling pathways (epidermal growth factor - *Egf*, fibroblast growth factor - *Fgf*, platelet-derived growth factor - *Pdgf*) identified through pathway analysis, as well as gene expression data on commonly expressed kinases and phosphatases, are presented in Supplemental Tables S3–S6.

### Extracellular Matrix

**Figure 5:**
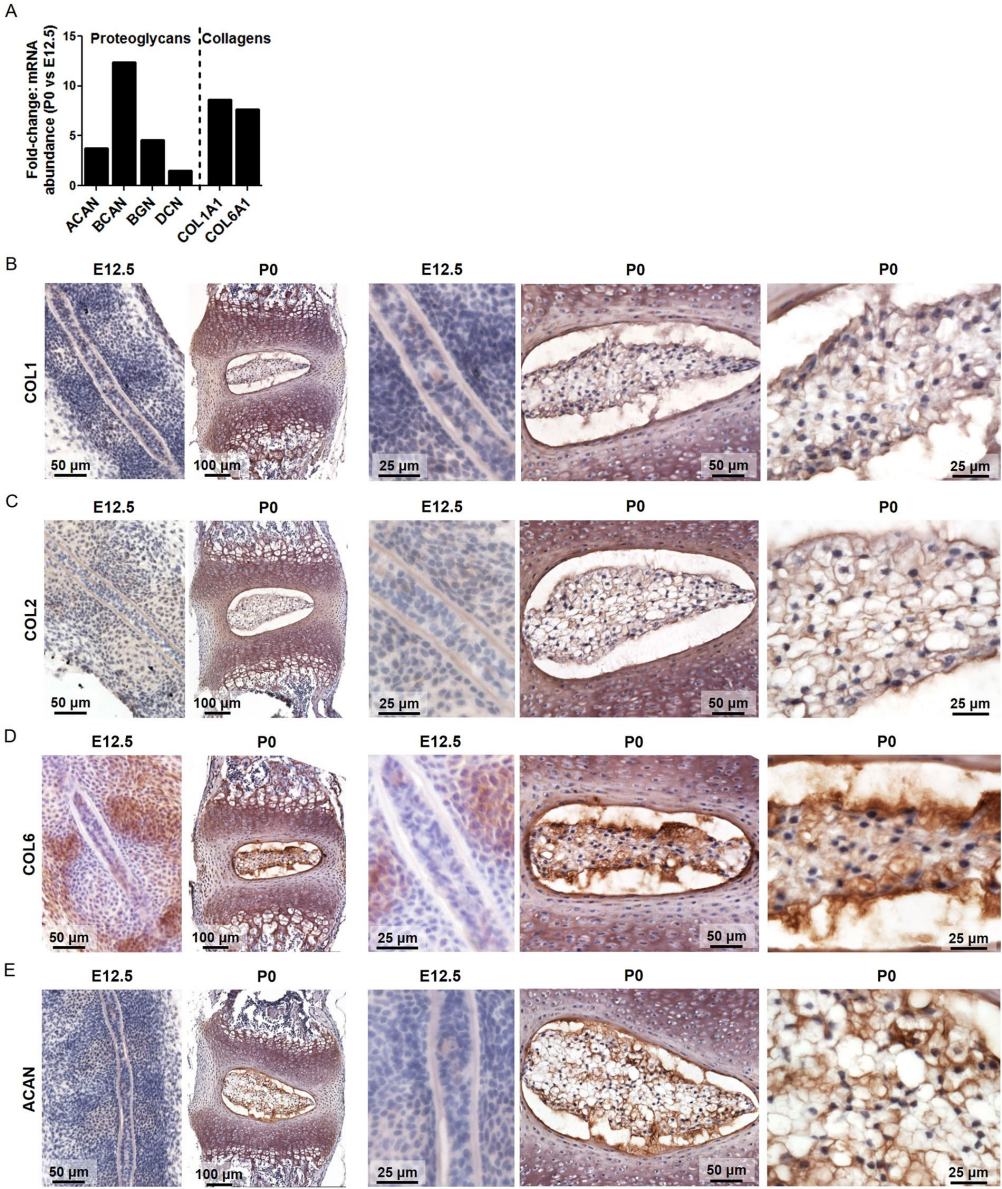
Fold-change in mRNA expression of P0 vs E12.5 NDCs and immunohistochemical analysis of key ECM molecules at E12.5 and P0. (**A**) Fold-change in mRNA expression of proteoglycans and collagens showing significant upregulation at P0. Protein staining for (**B**) COL1, (**C**) COL2, (**D**) COL6, and (**E**) ACAN, showing weak staining in non-cellular regions (core and sheath) of the E12.5 notochord and intense positive staining of the NP at P0. N = 4, all fold changes p < 0.05, P0 compared to E12.5, analyzed by DESeq2 and adjusted for false discovery rate. Refer to Fig. 2 for annotation of structural regions.

There was significantly higher mRNA abundance of ECM structural molecules, including proteoglycans (aggrecan (*Acan*); brevican (*Bcan*); biglycan (*Bgn*); decorin (*Dcn*)) and collagens (*Col1a1*, *Col6a1*), at P0 compared to E12.5 (Tables 4 and 5, Fig. 5A). Protein expression of COL1, COL2, COL6, and ACAN at each developmental stage was examined using immunohistochemistry (Fig. 5B–E). Overall, COL1, COL6, and ACAN showed diffuse staining in non-cellular regions (core and sheath) of the notochord at E12.5. At P0, COL6, and ACAN exhibited diffuse positive protein staining throughout the NP ECM, with intense staining in the outer boundary of the NP (Fig. 5D,E). Diffuse positive COL1 staining was present throughout the NP at P0 (Fig. 5B). There was no significant difference in the mRNA abundance of *Col2a1* between E12.5 and P0, and IHC revealed diffuse protein expression of COL2 in the notochord and NP at each respective developmental stage (Fig. 5C).

**Table 4:** Fold-change in mRNA expression of proteoglycan genes at P0 compared to E12.5.

Gene	Name	Fold Change in Expression	p value
		P0 vs E12.5	
*Acan*	Aggrecan	3.78	4.14E-03
*Agrn*	Agrin	−7.00	1.56E-14
*Bcan*	Brevican	12.39	2.25E-04
*Bgn*	Biglycan	4.56	4.11E-08
*Epyc*	Epiphycan	−7.46	3.39E-04
*Fmod*	Fibromodulin	4.91	5.29E-05
*Gpc2*	Glypican-2	−8.12	1.07E-16
*Gpc3*	Glypican-3	−4.66	2.95E-06
*Ncan*	Neurocan	−2.59	1.35E-02
*Omd*	Osteomodulin	5.28	2.45E-02
*Prelp*	Prolargin	2.66	1.92E-02
*Sdc1*	Syndecan-1	−4.63	1.02E-10
*Sdc3*	Syndecan-3	2.70	5.15E-03
*Sdc4*	Syndecan-4	3.80	2.80E-05
*Vcan*	Versican	−20.00	1.37E-26

N = 4, Differential expression analyzed by DESeq2 and p values adjusted for false discovery rate.

**Table 5:** Fold-change in mRNA expression of collagen genes at P0 compared to E12.5.

Gene	Name	Fold Change in Expression P0 vs E12.5	p value
*Col1a1*	Collagen I alpha 1	8.65	1.20E-07
*Col1a2*	Collagen I alpha 2	5.54	7.67E-09
*Col3a1*	Collagen III alpha 1	−3.87	4.94E-05
*Col4a1*	Collagen IV alpha 1	−6.88	3.44E-11
*Col4a2*	Collagen IV alpha 2	−5.70	1.97E-09
*Col4a5*	Collagen IV alpha 5	−6.18	1.63E-16
*Col4a6*	Collagen IV alpha 6	−5.10	1.48E-03
*Col9a1*	Collagen IX alpha 1	−7.31	4.85E-09
*Col9a2*	Collagen IX alpha 2	−20.46	6.12E-26
*Col9a3*	Collagen IX alpha 3	−4.34	1.98E-05
*Col5a3*	Collagen V alpha 3	4.29	1.07E-03
*Col6a1*	Collagen VI alpha 1	7.68	8.97E-08
*Col6a2*	Collagen VI alpha 2	5.80	2.15E-05
*Col10a1*	Collagen X alpha 1	8.88	2.41E-12
*Col11a1*	Collagen XI alpha 1	−3.33	1.67E-03
*Col12a1*	Collagen XII alpha 1	−2.79	3.45E-04
*Col14a1*	Collagen XIV alpha 1	−4.59	6.75E-11
*Col19a1*	Collagen XIX alpha 1	−4.94	1.79E-05
*Col17a1*	Collagen XVII alpha 1	−3.42	3.93E-03
*Col18a1*	Collagen XVIII alpha 1	−5.20	8.01E-07
*Col20a1*	Collagen XX alpha 1	−4.20	1.12E-04
*Col22a1*	Collagen XXII alpha 1	4.18	2.15E-05
*Col23a1*	Collagen XXIII alpha 1	−4.69	8.56E-06
*Col25a1*	Collagen XXV alpha 1	−4.18	3.20E-05
*Col26a1*	Collagen XXVI alpha 1	−9.80	1.02E-13
*Col27a1*	Collagen XXVII alpha 1	−6.74	5.99E-09
*Col28a1*	Collagen XXVIII alpha 1	5.28	1.64E-02

N = 4, Differential expression analyzed by DESeq2 and p values adjusted for false discovery rate.

With respect to surrounding tissue structures, at E12.5, condensations in the regions of the future discs exhibited positive staining for COL1 and COL6 protein (Fig. 5B,D). At P0, there was positive staining for COL2 protein in the inner AF, cartilaginous epiphyses, growth plates, and primary spongiosa (Fig. 5C). COL1 protein staining was observed in the AF and ossified regions of the vertebral bodies (Fig. 5B). COL6 and ACAN staining were present in the growth plates (Fig. 5D,E).

### Nucleus Pulposus Cell-Specific Markers

Finally, we examined differential mRNA expression of molecules considered to be specific markers of the NP cell phenotype^37^. Several such markers exhibited stable mRNA expression across the E12.5 to P0 developmental window, including brachyury (*T*), keratins 8 and 18 (*Krt8*, *Krt18*), and hypoxia-induced factor (*Hif1a*) (Table 6). Other NP markers exhibited significant changes in mRNA expression from E12.5 to P0, including cluster of differentiation 24 (*Cd24*, 2.48-fold increase), keratin 19 (*Krt19*, 2.9-fold increase), carbonic anhydrase 3 (*Car3*, 8.3-fold increase), carbonic anhydrase 12 (*Car12*, 4.6-fold decrease), and vimentin (*Vim*, 5.7-fold increase) (Table 6).

**Table 6:** Fold-change in mRNA expression of putative NP marker genes at P0 compared to E12.5.

Gene	Name	Fold Change in Expression P0 vs E12.5	p value
*Car12*	Carbonic anhydrase 12	−4.46	0.006
*Car3*	Carbonic anhydrase 3	7.41	3.79E-07
*Cd24*	Cluster of differentiation 24	2.48	0.003
*Krt19*	Cytokeratin 19	3.24	0.003
*Sostdc1*	Sclerostin domain-containing protein 1	9.09	1.83E-10
*Vim*	Vimentin	5.22	2.91E-08

N = 4, Differential expression analyzed by DESeq2 and p values adjusted for false discovery rate.

## Discussion

Recapitulation of developmental signaling responsible for embryonic NP formation represents a promising strategy for regenerating the intervertebral discs. During embryonic development, the cells of the notochord function both as a source of secreted signaling molecules to regulate the patterning of the discs and surrounding tissues and as the direct progenitors of NP cells. Notochordal NP cells from juvenile discs have been demonstrated to possess anabolic disease-modifying effects^40–45^, and thus, the application of notochord-derived factors in disc regeneration strategies has received considerable interest. In this study, we advance the molecular-level understanding of notochord cell function and disc development by using RNA-Seq to define the global changes in mRNA expression of NDCs at key stages of embryonic disc formation. It should be emphasized that the data analysis presented here, together with the immunohistochemical validation of a small number of molecules is a first step, and future work will delve further into specific pathways and gene families responsible for formation of the intervertebral discs. We will build on these results by extending RNA-Seq studies to additional pre- and postnatal developmental stages.

In the last decade, there have been important advances in our understanding of intervertebral disc development. Fate mapping studies in mice have provided conclusive evidence of the developmental origin of NP cells^16, 17^ and support the premise that these cells are the direct progenitors of adult NP cells. While these fate-mapping studies do not conclusively prove the notochordal origin of all NP cells in higher species, including humans, transcriptome analysis has demonstrated that adult human and bovine NP cells do highly express some notochordal cell-associated molecules^46, 47^. With respect to molecular regulation of embryonic disc formation, Shh and TGF-β signaling have both been established as indispensable for normal disc development^48–50^. SHH is a long range morphogen, expressed at various stages of embryonic development by cells of the primitive node, notochord, the neural tube floor plate, and posterior limb bud, which regulates spatial patterning, cell survival, and proliferation in both the axial skeleton and central nervous system^6, 14^. Shh signaling is critical for early embryonic patterning of the discs^50, 51^ and disruption of Shh signaling in mouse embryos through removal of Smo from the notochord and floor plate results in reduced NP size and absence of AF lamellar structure^50^. SHH is also required for formation of the sheath surrounding the notochord, and in its absence, notochord cells migrate aberrantly throughout surrounding tissues^50^. It has also been shown that SHH expressed solely by notochordal cells is sufficient for disc formation^51^. Interestingly, if SHH is removed subsequent to sheath formation the discs develop normally, suggesting that SHH is of foremost importance in the early stages of embryonic disc development^50^. Our finding in the current study that Shh signaling is significantly diminished at P0 compared to E12.5, evidenced by lower mRNA abundance of the ligand, receptor, and downstream transcriptional regulators and confirmed via immunohistochemistry (Fig. 4B), is consistent with this early embryonic role. A previous study showed that there continues to be *Shh* mRNA expressed in the mouse NP at E15.5, which is mid-way through the notochord to NP transformation^52^. While our results suggest a significantly diminished role for Shh signaling in the NP following embryonic development, there is evidence for a continuing postnatal role for this pathway^53, 54^, although the exact nature of this role and its importance relative to other pathways for regenerative therapeutics remains to be elucidated.

TGF-β signaling is critical for embryonic formation of both the NP and AF^48, 55, 56^. In mice where *Tgfbr2* is specifically removed from type II collagen expressing cells, the NPs that form are abnormally small suggesting that TGF-β signaling is required to drive NP growth^55^. TGF-β has been localized to human notochord cells and was shown to increase in expression from 12 to 14 weeks gestation^57^. The presence of all TGF-β isoforms and type I and II receptors has been demonstrated in the adult mouse NP, with expression decreasing progressively with aging^7^. In the current study, we found an 11-fold increase in *Tgfb1* mRNA abundance at P0 relative to E12.5 with protein expression confirmed using immunohistochemistry at both ages, and a corresponding 4-fold increase in *Tgfbr2* mRNA abundance. These results support the previously described central role for TGF-β signaling in the late embryonic and early postnatal differentiation and growth of the disc^38, 58^.

While the Shh and TGF-β pathways are perhaps the best characterized with respect to disc development, our results also demonstrated differential expression of secreted signaling molecules in other pathways. IGFs are important regulators of proliferation and differentiation during development across multiple skeletal cell types, including chondrocytes and osteoblasts^59, 60^. In NDCs, *Igf1* and *Igf2* exhibited opposite expression patterns, exhibiting higher and lower mRNA abundance at P0 compared to E12.5, respectively (Table 3). In general, these findings are consistent with existing literature for other tissues where *Igf1* has been shown to increase in expression during postnatal growth and into adulthood while *Igf2* is highest during embryonic and fetal development^11^. *Igf1r*-deficient mice exhibit signs of accelerated disc degeneration, suggesting *Igf1* is required to maintain the healthy adult disc^10^. In culture studies, IGF-1 has been shown to exert anabolic and anti-apoptotic effects on disc cells^18, 20, 29, 30, 32, 61, 62^ and has also been shown to exert anabolic effects on disc cells *in vivo* ^31^.

The ECM components of the NP are important in supporting tissue structure and biomechanical function as well as for the binding and distribution of the many secreted growth factors that mediate tissue morphogenesis, homeostasis, repair, and remodeling. The ECM composition of the adult NP has been widely studied. The two components that are principally important for mechanical function (specifically, resisting compressive forces) are COL2 and ACAN. Both COL2 and ACAN proteins have been shown previously to be expressed in the NP during embryonic development^12, 63^. COL2 is prominent in the human fetal NP from ~12 weeks gestation with ACAN also present as both diffuse and focal extracellular deposits^12^. COL2 is critical for the notochord to NP transformation^64^: in *Col2*-deficient mice the notochord persists as a rod-like structure until birth and intervertebral discs do not form. Here, we show that *Col2a1* exhibits stable mRNA abundance as the notochord transforms into the NP, while *Acan* abundance is higher at P0 compared to E12.5. Protein for both ACAN and COL2 was detected immunohistochemically at both developmental stages. Interestingly, we found increased mRNA abundance of *Col1*, not typically associated with the healthy NP matrix, at P0 relative to E12.5. In the adult disc, *Col1* is predominantly confined to the outer annulus fibrosus^1^ except during aging and degeneration when the NP becomes more fibrous^63^. Consistent with our results, however, *Col1* mRNA and protein have been demonstrated previously in the developing notochord and NP in humans and mice^12, 54, 57, 65^. We also found significantly elevated *Col6* mRNA expression at P0 (Table 4, Fig. 5A). COL6 protein, shown to be present in the human fetal disc from 12 weeks gestation and to increase with postnatal development and into adulthood^63^, exhibits predominantly pericellular localization^8^ and may play an important role in cell mechanotransduction similar to articular cartilage^9^. We also found significantly increased mRNA abundance of the small leucine rich repeat proteoglycans (SLRPs) *Bgn*, *Fmod*, and *Prelp* at P0. These molecules have established roles in the regulation of ECM assembly through binding to collagen fibrils and growth factors such as TGF-β and IGF-1^66–68^.

Finally, we examined the abundance of putative NP phenotypic markers shown previously to be expressed by immature and adult NP cells^38, 41, 42, 61, 63^. The search for NP markers has attracted fervent interest over the past decade due to their potential utility in validating NP-lineage specific differentiation of stem cells and to facilitate NP-specific analyses of gene function in transgenic animal models^37, 46, 47, 67, 69^. Here, we identify several such markers that exhibited stable or increased expression across the E12.5 to P0 developmental window that may be the most faithful indicators of notochordal and early postnatal NP cell phenotypes.

A limitation of this study was that we analyzed NDCs assuming a homogeneous population. Previous work demonstrated heterogeneity within the mouse NP cell population and the associated ECM, which manifests during later stages of development and into adulthood as a central notochordal-rich region surrounded by a halo of GAG-rich matrix-containing cells that are more chondrocyte-like. While our histological observations at P0 (Fig. 2D,E) superficially suggest a homogeneous cell population, it may well be the case that at the molecular level, phenotypically distinct subpopulations of cells are already beginning to emerge at this early postnatal developmental stage. Future work will seek to establish the extent to which altered gene expression at P0, and at later stages of postnatal development, reflects progressive heterogeneity in NP cellularity as a consequence of differentiation. While whole-transcriptome profiling studies such as this one provide comprehensive and high-fidelity gene-level information across a specified developmental window, we can only speculate as to the specific function and relative importance of many of those genes in disc development. To answer these questions, we anticipate that the results presented here will motivate future tissue-specific knockdown or overexpression studies in transgenic animal models.

In addition to improving fundamental understanding of molecular regulation of embryonic disc formation, our long term goal is to use these results to develop improved biologic strategies for regenerating NP tissue. While direct application of notochordal cells in cell-based therapeutics is limited due to their prenatal source, it may be possible to reprogram more readily available therapeutic cell types such as mesenchymal or induced pluripotent stem cells to mimic the secretory profile of notochordal cells, enhancing their potential for NP-specific tissue reconstitution. A promising strategy may be to drive stem cells towards a mature biosynthetic NP cell phenotype through sequential exposure to specific growth factors. A similar paradigm has been used previously to derive cartilage cells from pluripotent stem cell-derived progenitors^69^, and the results of this study provide a preliminary roadmap towards achieving this for disc regeneration.

## Conclusions

We have conducted the first transcriptome-wide analysis of notochord-derived cells during embryonic intervertebral disc formation. Overall, our results suggest that the secretory profile of NDCs changes from one optimized to direct tissue patterning through secretion of morphogens such as SHH at E12.5, to one focused on regulating cell differentiation, proliferation, and tissue growth through increased secretion of growth factors such as TGF-β1 and IGF-1 at P0 combined with increased synthesis of ECM molecules required to support the structure and mechanical function of the NP.

## Electronic supplementary material

Supplemental Information
