# Supplementary material for: Whole Transcriptome Analysis of Notochord-Derived Cells during Embryonic Formation of the Nucleus Pulposus
Source: Sci Rep. 2017 Sep 5;7:10504. doi: 10.1038/s41598-017-10692-5 (PMC5585380; doi:10.1038/s41598-017-10692-5)
Supplement: Supplementary file 1 — Supplemental Information [file 41598_2017_10692_MOESM1_ESM.pdf]

Supplemental Figures and Tables

Whole Transcriptome Analysis of Notochord-Derived Cells during Embryonic Formation of the Nucleus Pulposus

Sun H. Peck[1,2], Kendra K. McKee[3], John W. Tobias[4], Neil R. Malhotra[1,2], Brian D. Harfe[3], Lachlan J. Smith[1,2]*

[1]Department of Neurosurgery, Perelman School of Medicine, University of Pennsylvania

[2]Department of Orthopaedic Surgery, Perelman School of Medicine, University of Pennsylvania

[3]Department of Molecular Genetics and Microbiology, The Genetics Institute, College of Medicine, University of Florida

[4]Penn Genomics Analysis Core, University of Pennsylvania

*Correspondence:

Lachlan J. Smith, Ph.D.

Assistant Professor

Department of Neurosurgery

University of Pennsylvania

424 Stemmler Hall, 3450 Hamilton Walk

Philadelphia, PA 19104

Phone: 215 746 2169

Fax: 215 573 2133

Email: lachlans@mail.med.upenn.edu

Supplemental Figure S1: Representative images of negative controls for immunohistochemical analyses.

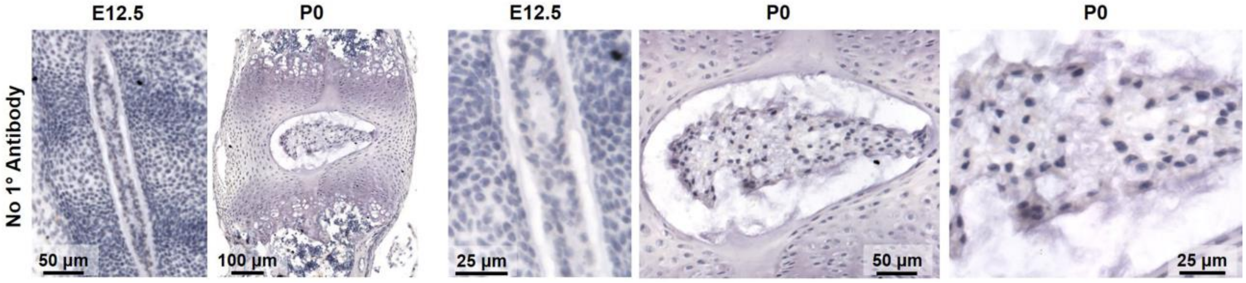

Supplemental Table S1: Primary antibodies used for immunohistochemistry.

**Antibody**	**Host**	**Company**	**Catalog Number**
SHH	Rabbit	Millipore	06-1106
PTCH1	Rabbit	Abcam	ab53715
TGF-β1	Rabbit	Abcam	ab66043
IGF-1	Rabbit	Abcam	ab40657
Aggrecan	Rabbit	Abcam	ab36861
Collagen 1	Rabbit	Abcam	ab34710
Collagen 2	Rabbit	Abcam	ab21291
Collagen 6	Rabbit	Fitzgerald	70R-Cr009x

Supplemental Table S2: Fold-change in mRNA expression of Wnt pathway genes at P0 compared to E12.5. N=4, Differential expression analyzed by DESeq2 and p values adjusted for false discovery rate.

Gene	Name	Fold Change in Expression P0 vs E12.5	p value
Axin2	axin 2	-6.95	8.30E-11
Bcl9	B-cell CLL/lymphoma 9	-2.81	4.03E-06
Ccnd1	cyclin D1	-4.30	1.45E-10
Cd44	CD44 molecule (Indian blood group)	13.20	1.09E-22
Cdh1	cadherin 1	-4.75	2.51E-06
Cdh3	cadherin 3	-53.52	1.23E-30
Dkk2	dickkopf WNT signaling pathway inhibitor 2	-10.18	6.44E-13
Frzb	frizzled-related protein	-2.83	8.79E-04
Fzd1	frizzled class receptor 1	-4.21	9.64E-07
Fzd2	frizzled class receptor 2	-8.11	7.97E-15
Fzd3	frizzled class receptor 3	-6.57	2.15E-17
Fzd4	frizzled class receptor 4	-2.30	1.55E-02
Fzd6	frizzled class receptor 6	-2.29	3.69E-02
Fzd7	frizzled class receptor 7	-2.02	3.86E-03
Fzd8	frizzled class receptor 8	-5.72	1.03E-07
Fzd9	frizzled class receptor 9	-5.55	1.05E-05
Fzd10	frizzled class receptor 10	-16.43	2.63E-07
Kremen1	kringle containing transmembrane protein 1	-3.38	3.19E-04
Kremen2	kringle containing transmembrane protein 2	-33.04	7.81E-15
Myc	v-myc avian myelocytomatosis viral oncogene homolog	-0.45	2.98E-04
Nlk	nemo-like kinase	-1.56	3.98E-02
Rarb	retinoic acid receptor, beta	-3.69	3.69E-04
Sfrp1	secreted frizzled-related protein 1	-14.51	3.57E-19
Sfrp2	secreted frizzled-related protein 2	-35.56	3.56E-27
Sfrp4	secreted frizzled-related protein 4	4.13	3.69E-02
Smo	smoothened, frizzled class receptor	-2.03	5.07E-03

Sox2	SRY-box 2	-7.20	1.71E-04
Sox4	SRY-box 4	-4.13	7.71E-11
Sox5	SRY-box 5	-3.84	1.25E-04
Sox7	SRY-box 7	-6.46	6.05E-06
Sox8	SRY-box 8	-2.37	2.42E-02
Sox9	SRY-box 9	-2.29	3.35E-02
Sox10	SRY-box 10	-11.52	6.36E-10
Sox11	SRY-box 11	-24.17	1.75E-24
Sox12	SRY-box 12	-3.47	1.85E-05
Sox13	SRY-box 13	-3.65	5.87E-05
Sox17	SRY-box 17	-10.50	5.01E-04
Tcf4	transcription factor 4	-3.79	6.44E-13
Tcf7l1	transcription factor 7-like 1 (T-cell specific, HMG-box)	-2.66	1.36E-02
Tle1	transducin like enhancer of split 1	-6.04	1.07E-20
Wnt1	wingless-type MMTV integration site family member 1	-6.42	1.25E-02
Wnt3	wingless-type MMTV integration site family member 3	-4.77	2.97E-03
Wnt6	wingless-type MMTV integration site family member 6	-3.25	2.30E-02
Wnt11	wingless-type MMTV integration site family member 11	-3.23	5.35E-04
Wnt16	wingless-type MMTV integration site family member 16	8.56	8.65E-06
Wnt2b	wingless-type MMTV integration site family member 2B	-2.67	6.65E-04
Wnt5a	wingless-type MMTV integration site family member 5A	-3.52	2.26E-05
Wnt7a	wingless-type MMTV integration site family member 7A	-5.75	8.30E-03
Wnt7b	wingless-type MMTV integration site family member 7B	-35.31	3.47E-13
Wnt9b	wingless-type MMTV integration site family member 9B	-6.71	3.81E-03

Supplemental Table S3: Fold-change in mRNA expression of EGF pathway genes at P0 compared to E12.5. N=4, Differential expression analyzed by DESeq2 and p values adjusted for false discovery rate.

Gene	Name	Fold Change in Expression P0 vs E12.5	p value
Elk1	ELK1, member of ETS oncogene family	-1.86	1.61E-02
Fos	FBJ murine osteosarcoma viral oncogene homolog	23.33	4.50E-12
Grb2	growth factor receptor bound protein 2	2.64	1.32E-04
Itpr1	inositol 1,4,5-trisphosphate receptor, type 1	-1.76	4.88E-02
Itpr2	inositol 1,4,5-trisphosphate receptor, type 2	2.10	2.58E-02
Itpr3	inositol 1,4,5-trisphosphate receptor, type 3	3.17	6.89E-03
Jak1	Janus kinase 1	2.77	3.88E-05
Prkca	protein kinase C, alpha	6.00	8.20E-22
Src	SRC proto-oncogene, non-receptor tyrosine kinase	-2.02	8.40E-03
Stat1	signal transducer and activator of transcription 1	8.94	5.36E-10
Stat3	signal transducer and activator of transcription 3 (acute-phase response factor)	2.89	6.16E-06

Supplemental Table S4: Fold-change in mRNA expression of FGF pathway genes at P0 compared to E12.5. N=4, Differential expression analyzed by DESeq2 and p values adjusted for false discovery rate.

Gene	Name	Fold Change in Expression P0 vs E12.5	p value
Creb3	cAMP responsive element binding protein 3	1.69	0.049
Creb5	cAMP responsive element binding protein 5	-5.33	6.00E-10
Fgf1	fibroblast growth factor 1 (acidic)	22.41	6.65E-24
Fgf2	fibroblast growth factor 2 (basic)	6.47	0.034
Fgf6	fibroblast growth factor 6	-17.27	0.017
Fgf10	fibroblast growth factor 10	-3.44	0.034
Fgf11	fibroblast growth factor 11	-2.55	0.015
Fgf15	fibroblast growth factor 15	-40.22	9.39E-06
Fgf18	fibroblast growth factor 18	-3.45	0.001
Fgfr1	fibroblast growth factor receptor 1	-2.13	0.012
Fgfr2	fibroblast growth factor receptor 2	-3.63	2.46E-07
Fgfr4	fibroblast growth factor receptor 4	-8.14	1.76E-04
Fgfrl1	fibroblast growth factor receptor-like 1	-2.64	0.012
Gab1	GRB2 associated binding protein 1	-1.64	0.031
Grb2	growth factor receptor bound protein 2	2.64	1.32E-04
Itpr1	inositol 1,4,5-trisphosphate receptor, type 1	-1.76	0.049
Ptpn6	protein tyrosine phosphatase, non-receptor type 6	19.03	1.32E-13
Stat3	signal transducer and activator of transcription 3 (acute-phase response factor)	2.89	6.16E-06

Supplemental Table S5: Fold-change in mRNA expression of PDGF pathway genes at P0 compared to E12.5. N=4, Differential expression analyzed by DESeq2 and p values adjusted for false discovery rate.

Gene	Name	Fold Change in Expression P0 vs E12.5	p value
Fos	FBJ murine osteosarcoma viral oncogene homolog	23.33	4.50E-12
Grb2	growth factor receptor bound protein 2	2.64	1.32E-04
Mapk3	mitogen-activated protein kinase 3	2.02	5.81E-04
Mras	muscle RAS oncogene homolog	1.99	4.69E-02
Myc	v-myc avian myelocytomatosis viral oncogene homolog	2.20	2.98E-04
Pdgfb	platelet derived growth factor subunit B	8.90	2.44E-05
Pdgfc	platelet derived growth factor C	-2.41	4.38E-03
Pdgfra	platelet derived growth factor receptor alpha	-3.93	1.05E-10
Pdgfrb	platelet derived growth factor receptor beta	-1.77	4.50E-02
Stat1	signal transducer and activator of transcription 1	8.94	5.36E-10
Stat3	signal transducer and activator of transcription 3 (acute-phase response factor)	2.89	6.16E-06
Synj1	synaptojanin 1	2.57	5.12E-05
Synj2	synaptojanin 2	-3.48	3.74E-06
Tyk2	tyrosine kinase 2	2.86	4.37E-04

Supplemental Table S6: Fold-change in mRNA expression of common kinase and phosphatase genes involved in pathway signaling at P0 compared to E12.5. N=4, Differential expression analyzed by DESeq2 and p values adjusted for false discovery rate.

Gene	Name	Fold Change in Expression P0 vs E12.5	p value
Inpp5d	inositol polyphosphate-5-phosphatase D	23.02	2.89E-12
Inpp5k	inositol polyphosphate-5-phosphatase K	2.53	2.64E-04
Jak1	Janus kinase 1	2.77	3.88E-05
Jak2	Janus kinase 2	1.98	3.85E-03
Jak3	Janus kinase 3	7.53	4.44E-07
Mapk3	mitogen-activated protein kinase 3	2.02	5.81E-04
Pik3c2b	phosphatidylinositol-4-phosphate 3-kinase catalytic subunit type 2 beta	-3.01	4.87E-04
Pik3cb	phosphatidylinositol-4,5-bisphosphate 3-kinase catalytic subunit beta	2.80	1.31E-04
Pik3cd	phosphatidylinositol-4,5-bisphosphate 3-kinase catalytic subunit delta	10.59	5.20E-19
Pik3cg	phosphatidylinositol-4,5-bisphosphate 3-kinase catalytic subunit gamma	24.45	1.63E-14
Pik3r3	phosphoinositide-3-kinase regulatory subunit 3	-5.95	1.04E-09
Pik3r5	phosphoinositide-3-kinase regulatory subunit 5	41.04	2.93E-05
Pik3r6	phosphoinositide-3-kinase regulatory subunit 6	15.16	1.34E-09
Prkacb	protein kinase, cAMP-dependent, beta catalytic subunit	2.62	1.82E-05
Prkar1b	protein kinase, cAMP-dependent, regulatory subunit type I beta	2.23	8.79E-03
Plcg1	phospholipase C gamma 1	-2.32	2.67E-05
Plcg2	phospholipase C gamma 2	12.86	3.15E-09
Ppm1j	protein phosphatase, Mg2+/Mn2+ dependent 1J	4.91	1.85E-03
Ppm1l	protein phosphatase, Mg2+/Mn2+ dependent 1L	-1.71	2.51E-02
Ppp2r2b	protein phosphatase 2 regulatory subunit B, beta	2.75	4.68E-04
Ppp2r5a	protein phosphatase 2 regulatory subunit B', alpha	3.67	1.13E-06
Ppp2r5b	protein phosphatase 2 regulatory subunit B', beta	2.16	1.02E-02

Prkca	protein kinase C, alpha	6.00	8.20E-22
Prkcb	protein kinase C, beta	9.95	4.10E-08
Src	SRC proto-oncogene, non-receptor tyrosine kinase	-2.02	8.40E-03
Tyk2	tyrosine kinase 2	2.86	4.37E-04
